# DFT Quantum-Chemical Modeling Molecular Structures of Cobalt Macrocyclic Complexes with Porphyrazine or Its Benzo-Derivatives and Two Oxygen Acido Ligands

**DOI:** 10.3390/ijms21239085

**Published:** 2020-11-29

**Authors:** Oleg V. Mikhailov, Denis V. Chachkov

**Affiliations:** 1 Kazan National Research Technological University, K. Marx Street 68, 420015 Kazan, Russia; 2 Kazan Department of Joint Supercomputer Center of Russian Academy of Sciences—Branch of Federal Scientific Center “Scientific Research Institute for System Analysis of the RAS”, Lobachevskii Street 2/31, 420111 Kazan, Russia; de2005c@gmail.com

**Keywords:** oxidation state VI, cobalt, macrocyclic chelate, oxo ligand, porphyrazine, benzo-derivative, DFT method

## Abstract

Based on the results of a quantum chemical calculation using the DFT method with the OPBE/TZVP and B3PW91/TZVP levels, the possibility of the existence of three cobalt heteroligand complexes containing in the inner coordination sphere porphyrazine, di[benzo]- and tetra[benzo]porphyrazine, and two oxygen (O^2−^) ions with probable oxidation state VI of Co, which is unknown for this element at the present time, was shown. Data on the structural parameters are presented. It was shown that CoN_4_ chelate nodes as well as all metal-chelate and non-chelate cycles in each of these complexes, were strictly planar. Besides, the bond angles formed by two donor nitrogen atoms and a Co atom were close or equal to 90°, while the bond angles formed by donor atoms N, Co, and O, in most cases, albeit insignificantly, differed from this value. Good agreement between the calculated data obtained using the above two versions of the DFT method was found. Standard thermodynamic parameters of formation (standard enthalpy Δ*H*^0^*_f_*_, 298_, entropy *S*^0^*_f_*_, 298_ and Gibbs’s energy Δ*G*^0^*_f_*_, 298_) for the indicated complexes were presented too.

## 1. Introduction

As is known, cobalt in its currently known chemical compounds can be in oxidation states from 0 up to V, wherein its highest oxidation state, namely IV and V, are uncharacteristic for the given element [1,2,3]. In particular, such compounds as CoO_2_ dioxide and cesium hexafluorocobaltate(IV) Cs_2_CoF_6_ [4,5], have been known for Co(IV) for a long time and for Co(V), sodium tetraoxocobaltates(V) Na_3_CoO_4_ and potassium K_3_CoO_4_ [6,7], and also a number of organometallic complexes [3,8,9,10]. An availability of Co(VI) was supposed in the matrix of cesium tetraoxoferrate(VI) (Cs_2_FeO_4_) as isomorphically replacing Fe(VI), and its identification was carried out by Mössbauer spectroscopy [11]; however, any compound with a stoichiometric chemical composition for a given oxidation state of cobalt was described neither in this work nor in other publications. By taking into account the theoretical considerations expressed in the introduction to the articles [12,13] devoted to Cu(IV) and Zn(IV) complexes with porphyrazine and its benzo-derivatives (in particular, *trans*-di[benzo]porphyrazine and tetra[benzo]porphyrazine) and one oxo ligand, we have every reason to believe that the given macrocyclic ligands in combination with two oxo ligands will also be able to form complexes with structural formulas [Co**L1**(O)_2_] (**I**), [Co**L2**(O)_2_] (**II**) and [Co**L3**(O)_2_] (**III**) (where **L1**^2−^, **L2**^2−^ and **L3**^2−^ are double deprotonated forms of porphyrazine H_2_**L1**, *trans*-di[benzo]porphyrazine H_2_**L2** and tetra[benzo]porphyrazine H_2_**L3**). These complexes are interesting in that, as it is easy to see from these formulas, in any of these complexes the cobalt atom is bonded to other atoms by eight chemical bonds, six of which are formed by the exchange mechanism (namely, two with each of two oxygen atoms and one with the each of two nitrogen atoms), and according to the generally accepted definition, the concept of “oxidation state”, the value of this parameter for a given atom of a 3d-element should be accepted equal to (+6). It should be specially noted that no mention of complexes of type **I**–**III** in the special literature devoted to (NNNN)-donor atomic macrocyclic ligands—porphyrins, porphyrazines and their derivatives [14,15,16,17,18,19,20,21,22], or anywhere else, could not be found. In this connection, the given article will be devoted to a theoretical analysis regarding the possibility of the existence in principle of such complexes with the use of modern methods of quantum chemistry, namely, the density functional theory (DFT) method. 
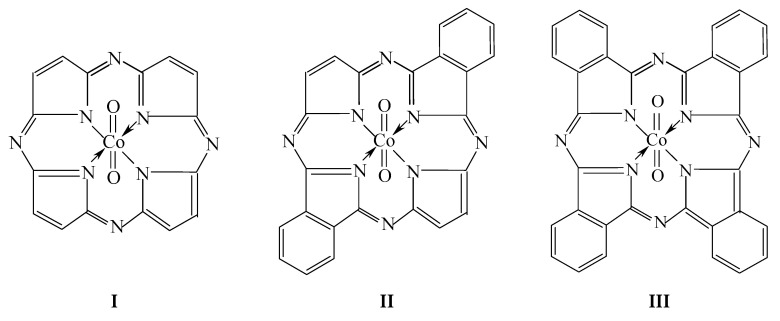


## 2. Results

**Figure 1:**
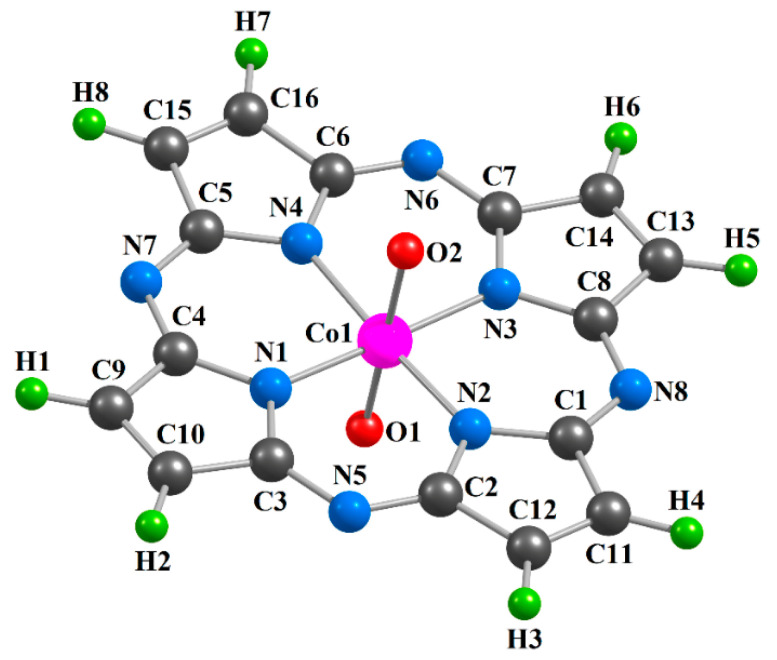
Molecular structure of the [Co**L1**(O)_2_] complex obtained as a result of DFT B3PW91/TZVP quantum-chemical calculation.

**Figure 2:**
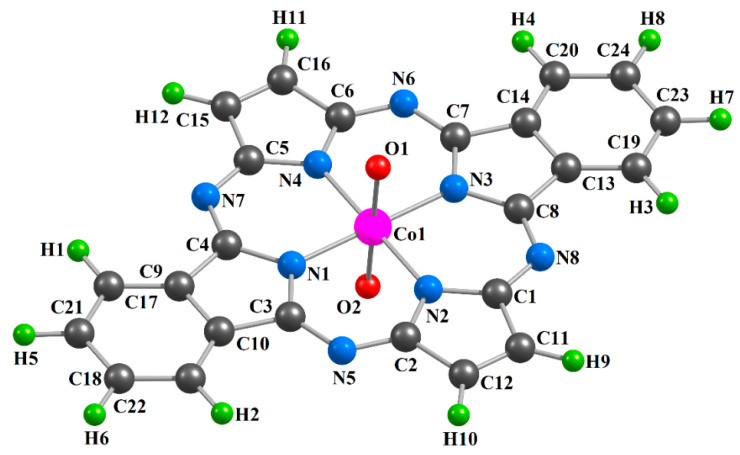
Molecular structure of the [Co**L2**(O)_2_] complex obtained as a result of DFT B3PW91/TZVP quantum-chemical calculation.

**Figure 3:**
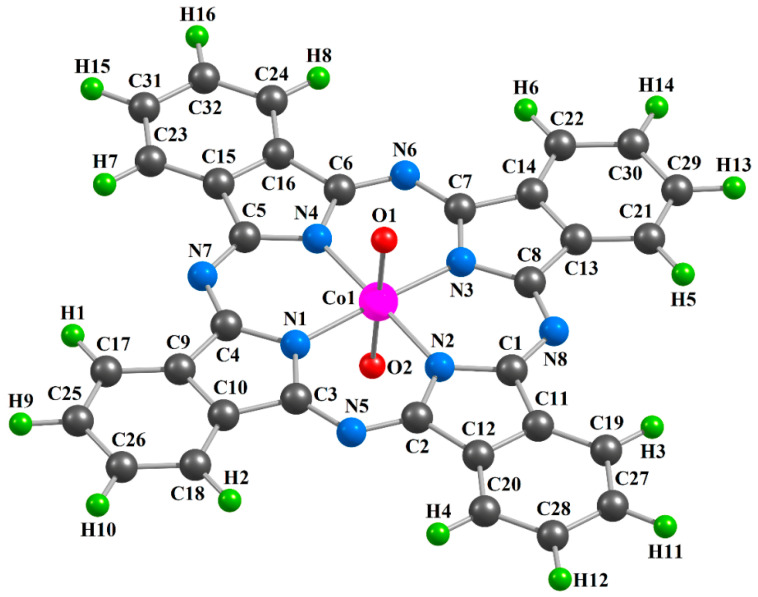
Molecular structure of the [Co**L3**(O)_2_] complex obtained as a result of DFT B3PW91/TZVP quantum-chemical calculation.

The chemical bond lengths between atoms and bond angles for the [Co**L1**(O)_2_] (**I**), [Co**L2**(O)_2_] (**II**) and [Co**L3**(O)_2_] (**III**) metal complexes calculated by the DFT OPBE/TZVP and DFT B3PW91/TZVP methods, are presented in the Table 1. The images of molecular structures of the given complexes obtained by the DFT B3PW91/TZVP method are shown in Figure 1, Figure 2 and Figure 3. The images of molecular structures of complexes under consideration obtained with the DFT OPBE/TZVP method are generally similar to those obtained with using the DFT B3PW91/TZVP method.

The values of the dipole electric moments for this complex, calculated using the OPBE/TZVP method, are 0.02 ([Co**L1**(O)_2_]), 0.01 ([Co**L2**(O)_2_]), and 0.00 ([Co**L3**(O)_2_]) Debye units; using the B3PW91/TZVP method, they are 0.27, 0.00 and 0.67 Debye units, respectively.

The most important data of NBO analysis, and namely the values of effective charges on Co, donor N and O atoms for heteroligand macrocyclic cobalt complexes under consideration obtained by the DFT OPBE/TZVP method, are presented in Table 2. For complete NBO analysis data, see Supplementary Materials, too.

**Figure 4:**
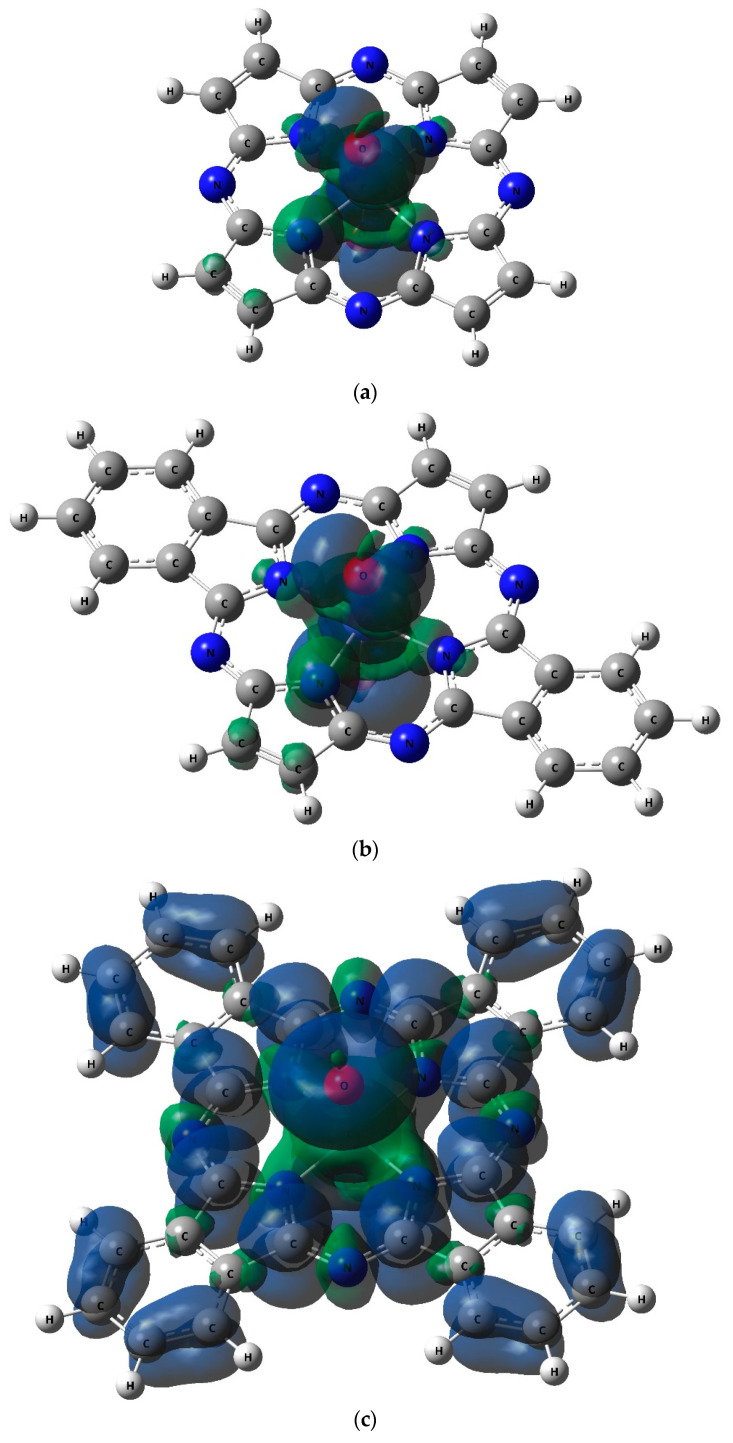
The pictures of spin density distribution in the [Co**L1**(O)_2_] (**a**), [Co**L2**(O)_2_] (**b**), and [Co**L3**(O)_2_] (**c**) complexes obtained by using the DFT OPBE/TZVP method.

The pictures of spin density distribution in the complexes under study are shown in Figure 4.

Standard thermodynamic parameters of formation (*ΔH*^0^_f, 298_, *S*^0^_f, 298_ and *ΔG*^0^_f, 298_) for [Co**L1**(O)_2_], [Co**L2**(O)_2_], and [Co**L3**(O)_2_] complexes are presented in Table 3. As may be seen, all these parameters are positive for each of the complexes under consideration.

## 3. Discussion

As can be seen from the data presented in Table 1, both versions of DFT methods used by us, on the whole, give similar values of key parameters of molecular structures for the chemical compounds under study. According to these data, all four 5-membered and 6-membered metal chelate rings as well as chelate nodes CoN_4_ and N_4_ groupings in them, are a strong plane or, at least, are practically plane because the sum of the bond angles in each of these structural fragments are exactly 540.0°, 720.0°, 360.0°, and 360.0°, respectively, or very close to these values (deviation from planarity is not more than 0.2°). Besides, all chelate and non-chelate rings are completely identical to each other in the sum of bond angles in them, but are slightly different between themselves in the lengths of bonds between the corresponding atoms and in the assortment of bond angles in them. In [Co**L1**(O)_2_] and [Co**L2**(O)_2_] complexes, however, both 5-membered and 6-membered rings are identical only in pairs because the assortment of bond angles in these pairs differ among themselves. This difference becomes quite understandable if we take into account only the pairwise equality of (NCoN) bond angles formed by the central metal atom Co with neighboring donor nitrogen atoms (Table 1). Unlike [Co**L1**(O)_2_] and [Co**L2**(O)_2_], in the [Co**L3**(O)_2_] complex, all these rings are fully identical between themselves (Table 1). According to DFT B3PW91/TZVP method data, all three complexes are characterized by a tetragonal-bipyramidal orientation of the donor nitrogen atoms relative to the 3*d*-element central atom whereas according to DFT OPBE/TZVP method data, this takes place only in the [Co**L3**(O)_2_] complex. Nevertheless, as a rule, the bond angles in the CoN_4_ chelate node between the nitrogen and cobalt atoms in the complexes under study are equal to each other and to 90°, or differ from this value by only 0.1°. The deviations (NCoO) in bond angles formed by nitrogen atoms in the chelate node, Co and oxygen atoms from 90° are more significant, sometimes reaching 5° (Table 1).

The bond lengths and bond angles listed above in [Co**L1**(O)_2_], [Co**L2**(O)_2_], and [Co**L3**(O)_2_] are quite typical for coordination compounds of 3*d*-elements containing **L1**^2−^, **L2**^2−^, and **L3**^2−^ as ligands (see, e.g., Supplementary Materials). It should be noted in this connection that within the framework of any of these structures, there are bonds between cobalt and oxygen atoms, the lengths of which (169–179 pm) correspond to double Co=O bonds, and bonds between cobalt and nitrogen atoms, the lengths of which (192–196 pm) correspond to a single Co–N bond. Note in this regard that the lengths of cobalt–nitrogen, carbon–nitrogen, and carbon–carbon bonds, as well as bond angles in the CoN_4_ chelate node and in metal chelate rings in the most bulky structures of these three complexes, namely [Co**L3**(O)_2_], are very close to the experimental values of the corresponding parameters for the complex of cobalt(II) with tetra [benzo]porphyrazine (phthalocyanine) [Co**L3**] found in [23,24].

As mentioned above, both the independent DFT methods used by us give rather close values of the key structural parameters for these complexes, but there are very significant differences on two points. On the one hand, according to the DFT OPBE/TZVP data, the bond angles formed by two oxygen and cobalt atoms (O1Co1O2) differ quite noticeably from 180°, while according to the DFT B3PW91/TZVP data, on the contrary, they are exactly 180°. On the other hand, that is interestingly the Co–O interatomic distances according to the OPBE/TZVP method, which are equal in each of the three complexes, while according to the DFT B3PW91/TZVP method, this equality occurs only in the case of [Co**L2**(O)_2_]. By taking into account this circumstance, one would expect that the electric moment of a dipole for [Co**L1**(O)_2_] and [Co**L2**(O)_2_], calculated using the DFT OPBE/TZVP method would be more significant than the electric moment of a dipole calculated using the DFT B3PW91/TZVP method. However, in fact, the opposite state of affairs takes place, because the values of the electric moment of a dipole for these complexes calculated by DFT OPBE/TZVP and DFT B3PW91/TZVP methods, are respectively 0.02 and 0.27 ([Co**L1**(O)_2_]), 0.01 and 0.00 ([Co**L2**(O)_2_]) Debye units. The reasons for this phenomenon remain yet unclear (be that as it may, none of them has a center of symmetry), but perhaps we are dealing here with an artifact or some calculation errors. However, in the case of [Co**L3**(O)_2_], where, according to the DFT OPBE/TZVP method data, the cobalt–oxygen bond lengths are the same (175.4 pm each), and according to the DFT B3PW91/TZVP method, they are significantly different (171.5 and 179.0 pm), one should expect a higher value of the dipole moment in the framework of the second of these methods, and it really turns out to be so (0.00 and 0.67 Debye units, respectively).

Earlier in the work [25], the molecular structure of the Co(IV) complex with porphyrazine and one axially oriented oxo-ligand of the formula [Co**L1**(O)] was calculated by the DFT OPBE/TZVP method; in this connection, it is possible to compare the key structural parameters of the [Co**L1**(O)] and [Co**L1**(O)_2_] complexes, calculated by this method. To begin with, according to [25], the CoN_4_ chelate node in the [Co**L1**(O)] complex, as well as in the [Co**L1**(O)_2_] complex, has a strictly planar structure, and the sets of (NCoN) bond angles in them is exactly the same; the groupings of donor nitrogen atoms N_4_ in both of these complexes are also strictly planar. The 5-membered chelate rings in both of these complexes are also strictly planar, while the 6-membered rings in them differ slightly from each other (in [Co**L1**(O)_2_], they are strictly flat, in [Co**L1**(O)], they have a small (1.2°) deviation from coplanarity [25]). The Co–N bond lengths in [Co**L1**(O)] are the same (190.4 pm each) and are somewhat shorter here than in [Co**L1**(O)_2_], whereas for the cobalt–oxygen bond lengths (170.1 pm and 168.9 pm, respectively) the opposite is true (Table 1). Since the [Co**L1**(O)] complex, as follows from the data [25], has a square-pyramidal structure, and the [Co**L1**(O)_2_] complex has an octahedral structure, then theoretically, the greatest difference between them should be expected for the bond angles formed of oxygen, cobalt, and donor nitrogen atoms, and this is indeed the case (these angles in the case of [Co**L1**(O)] are 96.5° and 95.3° [25], in the case of [Co**L1**(O)_2_], 85.1°, 90.0° and 94.9° (Table 1)). As can be seen from above, the key parameters of the molecular structures of heteroligand cobalt complexes containing porphyrazine and oxo ligands in the inner coordination sphere, but differing in the oxidation degree of the central atom (+4 in [Co**L1**(O)] and +6 in [Co**L1**(O)_2_]) are very similar to each other. Unfortunately, structural data on the [Co**L2**(O)] and [Co**L3**(O)] complexes obtained by quantum-chemical calculations by the DFT OPBE/TZVP method (as well as by any other calculation method) are still lacking in the literature, and, hence, a comparison between them and the [Co**L2**(O)_2_] and [Co**L3**(O)_2_] complexes under examination, respectively, does not seem possible.

As may be seen from Table 2, values of effective charges on Co atoms are considerably less than +6.000 ē which would be the case if all chemical bonds between Co, N, and O atoms would be ionic; the similar situation occurs for N donor atoms and O atoms, the values of charges on which are very different from −3.000 and −2.000 ē, respectively, too. This is evidence that a very high degree of electron density delocalization takes place in the each of the given coordination compounds.

According to the data of our calculations, the ground state of the [Co**L1**(O)_2_] and [Co**L2**(O)_2_] heteroligand complexes under study in the framework of the DFT OPBE/TZVP method is a spin doublet whereas the ground state of the [Co**L3**(O)_2_] one is a spin quartet. Besides, according to the data of this method, the nearest excited state (quartet in the case [Co**L1**(O)_2_] and [Co**L2**(O)_2_] and doublet in the case [Co**L3**(O)_2_]) has much higher energy (64.2, 33.8 and 39.9 kJ/mol, respectively). DFT B3PW91/TZVP give other data: the ground state of the [Co**L1**(O)_2_] and [Co**L3**(O)_2_] is a spin doublet, of the [Co**L2**(O)_2_] one is spin quartet; the nearest excited state is located higher than ground one, on 17.6, 48.3 and 55.9 kJ/mol, respectively. The difference in the values of the “energetic distances” in the ground and the nearest excited states given by these methods turns out to be very significant, but in any case, in our opinion, they allow us to make a conclusion that a spin crossover for such complexes should not have occurred. By taking into consideration the important fact that, as will be indicated further (see Section 4), the DFT OPBE/TZVP method in the case of 3*d* elements more adequately predicts the relative energy stabilities of high-spin and low-spin states than the DFT B3PW91/TZVP one, we consider it more correct to conclude that the ground state of [Co**L1**(O)_2_] and [Co**L2**(O)_2_] is namely a spin doublet, the ground state of [Co**L3**(O)_2_] is namely a spin quartet. An additional argument in favor of this conclusion is the values of <S**2> for these complexes (Table 2), each of which corresponds to the presence of precisely either one (in the case of [Co**L1**(O)_2_] and [Co**L2**(O)_2_]) or three (in the case of [Co**L3**(O)_2_]) unpaired electrons in the ground state and, therefore, *M_S_*= 2 and *M_S_*= 4, respectively. On the other hand, testing the wave functions of the ground state for stability within of this method using the STABLE = OPT procedure showed that, in all cases, the wave function did not show any attributes of instability.

## 4. Materials and Methods

As in our articles [12,13], and also in earlier ones, in particular [26,27,28,29,30], quantum-chemical calculations were performed by the DFT method with OPBE/TZVP level combining the common TZVP extended triple zeta split-valence basis set [31,32] and the OPBE non-hybrid functional [33,34]. As shown in [34,35,36,37,38], 3d elements more adequately predict the relative energy stabilities of high-spin and low-spin states, and reliably characterize the key geometric parameters of corresponding molecular structures. Moreover, for comparison, the other variant of the DFT method, namely with the B3PW91/TZVP level, which combining the TZVP and B3PW91 functional [39,40], was used in the given work; according to data [41], this has a minimal value of so-called “normal error” in comparison with other variants of the DFT method. This conclusion is in full harmony with the data of structural parameters of macrocyclic complexes of various 3*d*-elements with phthalocyanine obtained as a result of various DFT quantum-chemical calculations and in experiment (see Supplementary Materials). For comparison, the Supplementary Materials also presents the calculation data for the known complex of cobalt with phthalocyanine [Co**L3**], carried out using the DFTD wB97XD/TZVP method described in [42] and taking into account dispersion (Van der Waals) interactions; as it turned out, this method gives structural data close to those obtained by the DFT OPBE/TZVP and DFT B3PW91/TZVP methods. However, in comparison with them, it is much more costly in terms of the calculation time. That is why, [Co**L1**(O)_2_], [Co**L2**(O)_2_] and [Co**L3**(O)_2_] complexes under examination, were not calculated using the DFTD wB97XD/TZVP method.

Calculations were performed with the Gaussian09 program package [43]. The correspondence of the found stationary points to energy minima was proved in all cases by the calculation of second derivatives of energy with respect to atom coordinates; all equilibrium structures corresponding to minima of the potential energy surfaces had only real positive frequency values. When carrying out calculations within the framework of any of the above two variants of the DFT method (OPBE/TZVP as well as D3PW91/TZVP), we did not impose any symmetry restrictions; the structure with the *C*_1_ symmetry group was initially specified. Theoretically, Co(VI) must have 3*d*^3^ electronic configuration, and spin multiplicities 2 and 4 were considered for it. Among the structures optimized at these multiplicities, the lowest-lying structure was selected. Parameters of molecular structures with the given multiplicities were always calculated by unrestricted methods (UOPBE and UB3PW91, correspondingly). In addition, in all cases, the wave functions at the minimum point were checked for stability according to procedure STABLE = OPT in Gaussian. The standard thermodynamic parameters of formation (Δ*H*^0^*_f_*_, 298_, *S*^0^*_f_*_, 298_ and Δ*G*^0^*_f_*_, 298_) for the given macrocyclic metal chelates under examination were calculated using the method described in [44].

## 5. Conclusions

The data obtained using two various DFT methods, namely with OPBE/TZVP and B3PW91/TZVP levels and presented above in the given paper, unambiguously predicted the possibility of the existence of three novel cobalt complexes having [Co**L1**(O)_2_], [Co**L2**(O)_2_], and [Co**L3**(O)_2_] compositions where **L1**^2−^, **L2**^2−^, and **L3**^2−^ is a double deprotonated form of porphyrazine, *trans*-di[benzo]porphyrazine and tetra[benzo]porphyrazine, respectively. In each of them, there are six bonds formed by cobalt atoms with atoms having greater electronegativity in comparison with it, according to the exchange mechanism—two chemical bonds with nitrogen atoms, and four with oxygen atoms. The generally accepted definition of the term “oxidation degree” is as follows: “the oxidation degree is the charge in units of electron charge that would occur on the atom of a given element in a given chemical compound under the assumption that within the framework of each of the conditionally existing in this compound two-center two-electron chemical bonds formed by the exchange mechanism, there would be a complete transfer of electrons from the atom with less electronegativity to the atom with more electronegativity”. The oxidation degree of the each of central atoms in the metal complexes (i.e., Co) may be postulated equal to + 6. But since the oxidation state of any chemical element with a positive oxidation degree is determined as the modulus of oxidation degree and is displayed by the corresponding Roman numeral, in each of these compounds, the oxidation state of cobalt may be considered equal to VI. Naturally, the real charge on the Co atoms in the complexes under consideration differ significantly from the value of +6.00 ē, but given that the parameter is not connected with the above definition, in principle it cannot be used as a definition of the oxidation state [2]. Be that as it may, the results of our quantum-chemical calculation completely fit into the idea of a fairly high stability of those chemical compounds with a high oxidation state of the central atom, where the coordination polyhedron is formed from the most durable and difficult-to-oxidize acid ligands having atoms with the highest electronegativity (in our case, O^2−^). Note in this regard that, upon the introduction of the second oxo ligand into the inner coordination sphere of the square pyramidal complex [Co**L1**(O)] described in [25], the molecular structure of the resulting complex [Co**L1**(O)_2_] remains practically unchanged in comparison with that for [Co**L1**(O)_2_], both qualitatively and quantitatively.

As can be seen from the data presented in Table 3, the values of standard thermodynamic parameters of formation Δ*H*^0^*_f_*_, 298_, *S*^0^*_f_*_, 298_ and Δ*G*^0^*_f_*_, 298_ for the cobalt complexes of types **I**–**III** are positive and this is an indirect indication of their relatively low stability. This circumstance, however, is in no way a contraindication to attempts to obtain them, since it is known that there are a number of chemical compounds for which there is a similar situation with respect to standard thermodynamic characteristics, but which, nevertheless, were obtained experimentally in one way or another (f.e., boranes having general formula B_n_H_n+2_, dicarbon dinitride (cyanogen) C_2_N_2_ et al.) Moreover, according to the above data of a quantum-chemical calculation by the two independent DFT methods indicated above, the molecular structures of these complexes presented in Figure 1, Figure 2 and Figure 3 are quite stable). As for the possible synthesis of these chemical compounds, then, in principle, any of them could be obtained by exposure to very strong oxidizing agents, for example F_2_, KrF_2_, or of such an exotic oxidizer as difluoride dioxygen O_2_F_2_, on cobalt(II) complexes with the macrocyclic ligands **L1**, **L2** and **L3.** In particular, in the case of [Co**L3**(O)_2_], such a synthesis may be realized according to the general schemes (1–3).

[Co**L3**] + 2F_2_ + 4KOH → [Co**L3**(O)_2_] + 2H_2_O + 4KF (1)

[Co**L3**] + 2KrF_2_ + 4KOH → [Co**L3**(O)_2_] + 2Kr + 2H_2_O + 4KF (2)

[Co**L3**] + O_2_F_2_ + 2KOH →[Co**L3**(O)_2_] + 2H_2_O + 2KF (3)

It can be assumed that these macrocyclic compounds, if they will be obtained in experiment, will gain not only purely academic, but also very substantial practical interest and may be promising in a number of industry areas, in particular in catalysis, photonic, electronic, and electrochemical technologies. On the other hand, the complexes under study may be used as “precursors” in the various reactions of synthesis coordination compounds and, also, in the reactions of coordinated ligands owing to which, in principle, novel macrocyclic compounds may be obtained.

## Figures and Tables

**Table 1:** Bond lengths and bond angles in the nickel complexes of types **I, II,** and **III** calculated by OPBE/TZVP and B3PW91/TZVP methods.

Complex	[CoL1(O)_2_]		[CoL2(O)_2_]		[CoL3(O)_2_]	
Structural parameter	Calculated by DFT		Calculated by DFT		Calculated by DFT	
	OPBE/TZVP	B3PW91/TZVP	OPBE/TZVP	B3PW91/TZVP	OPBE/TZVP	B3PW91/TZVP
Co–N bond lengths in chelate node MN_4_, *pm*						
Co1N1	192.2	192.2	195.2	193.2	193.6	194.0
Co1N2	193.2	191.5	194.8	192.4	193.6	195.3
Co1N3	194.2	192.2	195.2	193.2	193.6	194.0
Co1N4	193.2	191.5	192.8	192.4	193.6	195.3
Co–O bond lengths, *pm*						
Co1O1	168.9	173.1	168.7	174.5	175.4	171.5
Co1O2	168.9	176.7	168.7	174.5	175.4	179.0
C–N bond lengths in 6-numbered chelate rings, *pm*						
N1C3	136.4	135.4	136.7	135.7	136.2	136.8
N1C4	136.4	135.4	136.6	135.7	136.2	136.8
N2C1	136.3	135.7	136.7	135.1	136.2	136.3
N2C2	136.2	135.7	136.7	135.1	136.2	136.3
N3C7	136.7	135.4	136.6	135.7	136.2	136.8
N3C8	136.7	135.4	136.7	135.7	136.2	136.8
N4C5	136.2	135.7	136.4	135.1	136.2	136.3
N4C6	136.3	135.7	136.4	135.1	136.2	136.3
N5C2	132.4	131.3	132.3	131.8	132.0	131.3
N5C3	132.4	132.7	131.9	131.8	132.0	131.3
N6C6	132.4	131.3	132.4	131.8	132.0	131.3
N6C7	132.3	132.7	131.9	131.8	132.0	131.3
N7C4	132.4	132.7	131.9	131.8	132.0	131.3
N7C5	132.4	131.3	132.4	131.8	132.0	131.3
N8C1	132.4	131.3	132.3	131.8	132.0	131.3
N8C8	132.3	132.7	131.9	131.8	132.0	131.3
C–C bond lengths in 5-numbered chelate ring (N1C4C9C10C3), *pm*						
C4C9	145.2	146.2	145.8	146.4	146.4	145.0
C9C10	135.7	134.4	140.2	139.4	139.9	139.7
C10C3	145.2	146.2	145.8	146.4	146.4	145.0
Bond angles in chelate node CoN_4_, *deg*						
(N1Co1N2)	90.1	90.0	89.9	90.0	90.0	90.0
(N2Co1N3)	89.9	89.9	89.9	90.0	90.0	90.0
(N3Co1N4)	89.9	90.0	90.1	90.0	90.0	90.0
(N4Co1N1)	90.1	89.9	90.1	90.0	90.0	90.0
Bond angles sum (*BAS*), *deg*	360.0	359.8	360.0	360.0	360.0	360.0
Non-bond angles between N atoms in N_4_ grouping, *deg*						
(N1N2N3)	90.0	90.2	90.2	90.2	90.0	89.6
(N2N3N4)	89.8	89.7	89.6	89.8	90.0	90.4
(N3N4N1)	90.0	90.2	90.6	90.2	90.0	89.6
(N4N1N2)	90.2	89.7	89.6	89.8	90.0	90.4
Non-bond angles sum (NBAS), *deg*	360.0	359.8	360.0	360.0	360.0	360.0
Bond angles in 6-numbered chelate ring (Co1N1C4N7C5N4), *deg*						
(Co1N1C4)	126.1	126.3	125.3	125.6	125.8	125.5
(N1C4N7)	128.3	127.9	128.4	128.4	128.5	128.3
(C4N7C5)	121.4	121.1	121.7	121.7	121.4	122.6
(N7C5N4)	128.1	128.4	128.3	128.0	128.5	128.1
(C5N4Co1)	126.1	126.3	126.2	126.3	125.8	125.5
(N4Co1N1)	90.0	89.9	90.1	90.0	90.0	90.0
Bond angles sum (BAS^61^), *deg*	720.0	719.9	720.0	720.0	720.0	720.0
Bond angles in 6-numbered chelate ring (Co1N4C6N6C7N3), *deg*						
(Co1N4C6)	126.1	126.3	126.2	126.3	125.8	125.6
(N4C6N6)	128.5	128.4	128.3	128.0	128.5	128.0
(C6N6C7)	121.4	121.1	121.7	121.7	121.4	122.6
(N6C7N3)	128.2	127.9	128.4	128.4	128.5	128.2
(C7N3Co1)	125.9	126.3	125.3	125.6	125.8	125.6
(N3Co1N4)	89.9	89.9	90.1	90.0	90.0	90.0
Bond angles sum (BAS^62^), *deg*	720.0	719.9	720.0	720.0	720.0	720.0
Bond angles in 5-numbered ring (C3N1C4C9C10), *deg*						
(C3N1C4)	107.8	107.3	109.3	108.8	108.4	108.9
(N1C4C9)	109.1	109.7	108.9	109.5	109.7	109.0
(C4C9C10)	107.0	106.6	106.4	106.1	106.1	106.6
(C9C10C3)	107.0	106.6	106.5	106.1	106.1	106.6
(C10C3N1)	109.1	109.7	108.9	109.5	109.7	108.9
Bond angles sum (BAS^51^), *deg*	540.0	539.9	540.0	540.0	540.0	540.0
Bond angles in 5-numbered ring (C1N2C2C12C11), *deg*						
(C1N2C2)	107.8	107.5	108.0	107.4	108.4	108.9
(N2C2C12)	109.2	109.4	108.9	109.8	109.7	109.1
(C2C12C11)	106.9	106.8	107.1	106.5	106.1	106.4
(C12C11C1)	107.0	106.8	107.1	106.5	106.1	106.4
(C11C1N2)	109.1	109.4	108.9	109.8	109.7	109.1
Bond angles sum (BAS^52^), *deg*	540.0	539.9	540.0	540.0	540.0	540.0
Bond angles between O, Co and N atoms, *deg*						
O1Co1N1	94.9	91.1	90.0	90.0	90.0	91.5
O1Co1N2	90.0	92.7	85.0	90.0	90.0	91.0
O1Co1N3	85.1	91.1	90.0	90.0	90.0	91.5
O1Co1N4	90.0	92.7	95.0	90.0	90.0	91.0
O2Co1N1	94.9	88.9	90.0	90.0	90.0	88.5
O2Co1N2	90.0	87.3	85.0	90.0	90.0	89.0
O2Co1N3	85.1	88.9	90.0	90.0	90.0	88.5
O2Co1N4	90.0	87.3	85.0	90.0	90.0	89.0
Bond angles between Co and two O atoms, *deg*						
O1Co1O2	170.1	180.0	169.9	180.0	180.0	180.0

**Table 2:** NBO analysis data for the cobalt complexes of types **I**, **II,** and **III**.

Complex	Effective Charge of Atom, in Units of Electron Charge (ē)				<S**2>
	Co1	N1 (N3)	N2 (N4)	O1 (O2)	
[Co**L1**(O)_2_]	+0.0647	−0.2989 (−0.2938)	−0.2905 (−0.2905)	−0.0895 (−0.0895)	0.7609
[Co**L2**(O)_2_]	+0.0859	−0.2699 (−0.2699)	−0.2982 (−0.3065)	−0.1673 (−0.1673)	0.7627
[Co**L3**(O)_2_]	+0.0881	−0.2669 (−02669)	−0.2669 (−02669)	−0.4071 (−0.4071)	3.7715

**Table 3:** Standard thermodynamic parameters of formation (*ΔH*^0^*_f_*_, 298_, *S*^0^*_f_*_, 298_ and *ΔG*^0^*_f_*_, 298_) for the cobalt complexes of types **I**–**III**.

Complex	Δ*H*^0^*_f_*_, 298_, kJ/mole	*S*^0^*_f_*_, 298_, J/mole ∙K	Δ*G*^0^*_f_*_, 298_, kJ/mole
[Co**L1**(O)_2_]	616.3	759.8	842.1
[Co**L2**(O)_2_]	567.7	949.8	814.5
[Co**L3**(O)_2_]	530.1	1168.9	789.3

## Supplementary Materials

Supplementary Materials can be found at https://www.mdpi.com/1422-0067/21/23/9085/s1.
