# Supplementary material for: DFT Quantum-Chemical Modeling Molecular Structures of Cobalt Macrocyclic Complexes with Porphyrazine or Its Benzo-Derivatives and Two Oxygen Acido Ligands
Source: Int J Mol Sci. 2020 Nov 29;21(23):9085. doi: 10.3390/ijms21239085 (PMC7730303; doi:10.3390/ijms21239085)
Supplement: Supplementary file 1 [file ijms-21-09085-s001.pdf]

Bonds lengths, valence and torsion angles in the Mn(II), Fe(II) and Co(II)) phthalocyanine metal chelates. The **bold font** in brackets specifies experimental values, regular font, calculated by DFT B3LYP 6-31G(d), OPBE/TZVP, B3PW91/TZVP and wB97XD/TZVP (first, second, third and fourth value, respectively) *.

M	Mn	Fe	Co
M–N bond lengths, *pm*			
(M1N1)	195.9; 195.5; 195.9; 194.1; (**193.9**)	193.8; 190.8; 194.0; 200.3; (**192.7**)	189.6; 191.5; 192.7; 192.9; (**191.0**)
(M1N2)	193.7; 193.7; 194.1; 195.8; (**193.8**)	193.8; 190.8; 194.0; 200.3; (**192.6**)	189.6; 191.5; 192.7; 192.9; (**191.0**)
(M1N3)	195.9; 195.5; 195.9; 194.1; (**193.9**)	193.8; 190.8; 194.0; 200.4; (**192.7**)	189.6; 191.5; 192.7; 192.9; (**191.0**)
(M1N4)	193.7; 193.7; 194.1; 195.8; (**193.8**)	193.8; 190.8; 194.0; 200.3; (**192.6**)	189.6; 191.5; 192.7; 192.9; (**191.0**)
C–N bond lengths, *pm*			
(N1C3)	138.5; 138.1; 137.5; 137.9; (**138.9**)	138.0; 138.2; 137.0; 136.2; (**138.1**)	138.8; 137.8; 137.0; 136.6; (**138.4**)
(N1C4)	138.5; 138.1; 137.5; 137.9; (**139.7**)	138.1; 138.3; 137.2; 136.2; (**137.5**)	138.8; 137.8; 137.0; 136.6; (**137.1**)
(N2C1)	139.5; 138.9; 138.4; 137.0; (**139.1**)	138.1; 138.3; 137.2; 136.2; (**137.5**)	138.8; 137.8; 137.0; 136.6; (**137.1**)
(N2C2)	139.5; 138.9; 138.4; 137.0; (**139.2**)	138.0; 138.3; 137.0; 136.1; (**138.2**)	138.8; 137.8; 137.0; 136.6; (**138.4**)
(N3C7)	138.5; 138.1; 137.5; 137.9; (**138.9**)	138.0; 138.2; 137.0; 136.1; (**138.1**)	138.8; 137.8; 137.0; 136.6; (**137.0**)
(N3C8)	138.5; 139.1; 137.5; 137.9; (**139.7**)	138.1; 138.3; 137.2; 136.1; (**137.5**)	138.8; 137.8; 137.0; 136.6; (**138.7**)
(N4C5)	139.5; 138.9; 138.4; 137.0; (**139.1**)	138.1; 138.3; 137.2; 136.1; (**137.5**)	138.8; 137.8; 137.0; 136.6; (**138.0**)
(N4C6)	139.5; 138.9; 138.4; 137.0; (**139.2**)	138.0; 138.2; 137.0; 136.2; (**138.2**)	138.8; 137.8; 137.0; 136.6; (**138.0**)
(N5C2)	131.2; 131.1; 130.6; 132.3; (**131.4**)	132.2; 131.9; 131.6; 132.3; (**132.1**)	131.9; 131.7; 131.4; 131.2; (**131.9**)
(N5C3)	133.1; 132.7; 132.3; 130.4; (**132.5**)	132.2; 131.9; 131.6; 132.2; (**132.2**)	131.9; 131.7; 131.4; 131.2; (**132.6**)
(N6C6)	131.2; 131.1; 130.6; 132.3; (**131.4**)	132.2; 131.9; 131.6; 132.1; (**132.1**)	131.9; 131.7; 131.4; 131.2; (**131.9**)
(N6C7)	133.1; 132.7; 132.3; 130.4; (**132.5**)	132.2; 131.9; 131.6; 132.4; (**132.2**)	131.9; 131.7; 131.4; 131.2; (**132.6**)
(N7C4)	133.1; 132.7; 132.3; 130.4; (**132.4**)	132.2; 131.9; 131.6; 132.2; (**132.0**)	131.9; 131.7; 131.4; 131.2; (**132.3**)
(N7C5)	131.2; 132.8; 130.6; 132.3; (**132.8**)	132.2; 131.9; 131.6; 132.3; (**132.4**)	131.9; 131.7; 131.4; 131.2; (**132.5**)
(N8C1)	131.2; 131.1; 130.6; 132.3; (**132.8**)	132.2; 131.9; 131.6; 132.1; (**132.4**)	131.9; 131.7; 131.4; 131.2; (**132.3**)
(N8C8)	133.1; 132.7; 132.3; 130.4; (**132.4**)	132.2; 131.9; 131.6; 132.4; (**132.0**)	131.9; 131.7; 131.4; 131.2; (**132.5**)

C–C bond lengths, *pm*			
(C9C10)	141.2; 141.0; 140.5; 139.1; (**140.8**)	140.4; 140.0; 139.9; 140.0; (**139.0**)	139.9; 140.1; 139.7; 139.2; (**139.2**)
(C11C12)	140.3; 140.2; 139.7; 139.9; (**140.7**)	140.4; 140.0; 139.9; 140.0; (**139.3**)	139.9; 140.1; 139.7; 139.2; (**139.8**)
(C13C14)	141.2; 141.0; 140.5; 139.1; (**140.8**)	140.4; 140.0; 139.9; 140.1; (**139.0**)	139.9; 140.1; 139.7; 139.2; (**139.2**)
(C15C16)	140.3; 140.2; 139.7; 139.9; (**140.7**)	140.4; 140.0; 139.9; 140.0; (**139.3**)	139.9; 140.1; 139.7; 139.2; (**139.8**)
(C9C17)	140.1; 139.9; 139.4; 138.6; (**139.2**)	139.6; 139.2; 139.0; 138.7; (**139.5**)	139.2; 139.5; 138.9; 138.8; (**139.3**)
(C17C25)	138.8; 138.8; 138.2; 138.6; (**139.4**)	139.3; 139.7; 138.7; 138.5; (**138.7**)	139.8; 139.2; 138.7; 138.4; (**139.4**)
(C25C26)	141.5; 141.0; 140.7; 139.8; (**140.9**)	140.9; 140.1; 140.2; 140.0; (**139.4**)	140.4; 140.5; 140.2; 140.1; (**139.1**)
(C26C18)	138.8; 138.8; 138.2; 138.6; (**139.6**)	139.3; 139.7; 138.7; 138.5; (**139.7**)	139.8; 139.2; 138.7; 138.4; (**139.4**)
(C18C10)	140.1; 139.9; 139.4; 138.6; (**140.0**)	139.6; 139.2; 138.9; 138.7; (**139.4**)	139.2; 139.5; 138.9; 138.8; (**139.0**)
C–H bond lengths, *pm*			
(C17H1)	108.5; 108.8; 108.3; 108.2; (**109.5**)	108.5; 108.9; 108.3; 108.2; (**–**)	108.5; 108.8; 108.3; 108.2; (**95.3**)
(C25H9)	108.7; 109.0; 108.4; 108.3; (**107.8**)	108.7; 109.0; 108.4; 108.3; (**–**)	108.6; 109.0; 108.4; 108.3; (**95.8**)
(C26H10)	108.7; 109.0; 108.4; 108.3; (**109.4**)	108.7; 109.0; 108.4; 108.3; (**–**)	108.6; 109.0; 108.4; 108.3; (**96.3**)
(C18H2)	108.5; 108.8; 108.3; 108.2; (**108.0**)	108.5; 108.9; 108.3; 108.2; (**–**)	108.5; 108.8; 108.3; 108.2; (**95.1**)
∠NMN bond angles in the MN_4 chelate node, *deg*			
(N1M1N4)	90.0; 90.0; 90.0; 90.0; (**91.3**)	90.0; 90.0; 89.9; 90.0; (**90.9**)	90.0; 90.0; 90.0; 90.0; (**90.0**)
(N4M1N3)	90.0; 90.0; 90.0; 90.0; (**88.7**)	90.0; 90.0; 90.1; 90.0; (**89.1**)	90.0; 90.0; 90.0; 90.0; (**90.0**)
(N3M1N2)	90.0; 90.0; 90.0; 90.0; (**91.3**)	90.0; 90.0; 89.9; 90.0; (**90.9**)	90.0; 90.0; 90.0; 90.0; (**90.0**)
(N2M1N1)	90.0; 90.0; 90.0; 90.0; (**88.7**)	90.0; 90.0; 90.1; 90.0; (**89.1**)	90.0; 90.0; 90.0; 90.0; (**90.0**)
VAS	360.0; 360.0; 360.0; 360.0; (**360.0**)	360.0; 360.0; 360.0; 360.0; (**360.0**)	360.0; 360.0; 360.0; 360.0; (**360.0**)
∠NNN non-bond angles in the MN_4 chelate node, *deg*			
(N1N4N3)	90.7; 90.5; 90.5; 89.5; (**90.5**)	90.0; 90.0; 90.0; 90.0; (**90.0**)	90.0; 90.0; 90.0; 90.0; (**90.0**)
(N4N3N2)	89.3; 89.5; 89.5; 90.5; (**89.5**)	90.0; 90.0; 90.0; 90.0; (**90.0**)	90.0; 90.0; 90.0; 90.0; (**90.0**)
(N3N2N1)	90.7; 90.5; 90.0; 89.5; (**90.5**)	90.0; 90.0; 90.0; 90.0; (**90.0**)	90.0; 90.0; 90.0; 90.0; (**90.0**)
(N2N1N4)	89.3; 90.5; 89.5; 90.5; (**89.5**)	90.0; 90.0; 90.0; 90.0; (**90.0**)	90.0; 90.0; 90.0; 90.0; (**90.0**)
NVAS	360.0; 360.0; 360.0; (**360.0**)	360.0; 360.0; 360.0; 360.0; (**360.0**)	360.0; 360.0; 360.0; 360.0; (**360.0**)

Bond angles in the 6-numbered ring (M1N1C4N7C5N4), *deg*			
(N1M1N4)	90.0; 90.0; 90.0; 90.0; (**91.3**)	90.0; 90.0; 89.9; 90.0; (**90.9**)	90.0; 90.0; 90.0; 90.0; (**90.0**)
(M1N4C5)	126.2; 126.1; 126.0; 125.6; (**125.2**)	126.3; 126.7; 126.0; 124.7; (**125.4**)	127.0; 126.4; 126.1; 126.1; (**127.8**)
(N4C5N7)	127.5; 127.6; 127.5; 127.3; (**127.9**)	127.5; 127.6; 127.5; 127.6; (**128.0**)	127.6; 127.8; 127.6; 127.7; (**126.2**)
(C5N7C4)	123.1; 123.0; 123.6; 123.7; (**122.7**)	122.5; 121.4; 123.0; 125.4; (**122.2**)	120.8; 121.5; 122.5; 122.6; (**121.1**)
(N7C4N1)	127.2; 127.4; 127.2; 127.5; (**127.7**)	127.5; 127.6; 127.5; 127.5; (**127.9**)	127.6; 127.8; 127.6; 127.6; (**128.4**)
(C4N1M1)	125.9; 125.8; 125.7; 125.9; (**125.2**)	126.2; 126.7; 126.0; 124.8; (**125.5**)	127.0; 126.4; 126.1; 126.0; (**125.8**)
VAS[1]	719.9; 719.9; 720.0; 720.0; (**720.0**)	720.0; 720.0; 719.9; 720.0; (**719.9**)	720.0; 719.9; 719.9; 720.0; (**719.3**)
Bond angles in the 5-numbered ring (C3N1C4C9C10), *deg*			
(C3N1C4)	108.2; 108.4; 108.7; 108.2; (**107.5**)	107.6; 106.6; 108.1; 110.4; (**107.3**)	106.1; 107.1; 107.7; 107.9; (**107.3**)
(N1C4C9)	109.2; 109.1; 108.9; 109.1; (**109.5**)	109.8; 110.4; 109.5; 108.1; (**110.0**)	110.6; 110.1; 109.8; 109.7; (**109.3**)
(C4C9C10)	106.7; 106.7; 106.7; 106.8; (**106.6**)	106.4; 106.3; 106.4; 106.7; (**106.6**)	106.3; 106.3; 106.3; 106.3; (**106.2**)
(C9C10C3)	106.7; 106.7; 106.7; 106.8; (**107.0**)	106.4; 106.3; 106.5; 106.7; (**106.5**)	106.3; 106.3; 106.3; 106.4; (**106.5**)
(C10C3N1)	109.2; 109.1; 108.9; 109.1; (**109.3**)	109.8; 110.4; 109.5; 109.7; (**109.6**)	110.6; 110.1; 109.8; 110.0; (**108.2**)
VAS[2]	540.0; 540.0; 539.9; 540.0; (**539.9**)	540.0; 540.0; 540.0; 540.0; (**540.0**)	539.9; 539.9; 539.9; 540.0; (**537.5**)
Bond angles in the 6-numbered ring (C9C10C18C26C25C17), *deg*			
(C9C10C18)	121.0; 120.1; 121.0; 121.3; (**120.8**)	121.2; 121.2; 121.1; 121.1; (**121.4**)	121.4; 121.3; 121.3; 121.4; (**119.6**)
(C10C18C26)	117.7; 117.8; 117.7; 117.5; (**117.5**)	117.6; 117.7; 117.6; 117.7; (**117.0**)	117.5; 117.5; 117.5; 117.4; (**119.2**)
(C18C26C25)	121.3; 121.3; 121.3; 121.2; (**121.3**)	121.2; 121.1; 121.3; 121.2; (**121.2**)	121.1; 121.2; 121.2; 121.2; (**119.4**)
(C26C25C17)	121.3; 121.3; 121.3; 121.2; (**121.3**)	121.2; 121.1; 121.1; 121.2; (**121.8**)	121.1; 121.2; 121.2; 121.2; (**120.0**)
(C25C17C9)	117.7; 117.8; 117.7; 117.5; (**117.3**)	117.6; 117.7; 117.6; 117.7; (**116.9**)	117.5; 117.5; 117.5; 117.4; (**119.7**)
(C17C9C10)	121.0; 121.0; 121.0; 121.3; (**121.8**)	121.2; 121.2; 121.3; 121.1; (**121.7**)	121.4; 121.3; 121.3; 121.4; (**119.6**)
VAS[3]	720.0; 719.3; 720.0; 720.0; (**720.0**)	720.0; 720.0; 720.0; 720.0; (**720.0**)	720.0; 720.0; 720.0; 720.0; (**717.5**)
Selected torsion angles, *deg*			
(M1N1C4N7)	0.0; 0.0; 0.0; 0.0; 0.0; (**1.1**)	0.0; 0.0; 0.0; 0.0; (**0.0**)	0.0; 0.0; 0.0; 0.0; (**11.0**)
(N1C4C9C17)	180.0; 180.0; 180.0; 180.0; (**176.9**)	180.0; 180.0; 180.0; 180.0; (**178.8**)	180.0; 180.0; 180.0; 180.0; (**175.8**)
(N1C3C10C18)	180.0; 180.0; 180.0; 180.0; (**177.3**)	180.0; 180.0; 180.0; 180.0; (**178.6**)	180.0; 180.0; 180.0; 180.0; (**157.8**)
(N7C4C9C17)	0.0; 0.0; 0.0; 0.0; (**1.9**)	0.0; 0.0; 0.0; 0.0; (**0.0**)	0.0; 0.0; 0.0; 0.0; (**3.4**)

(N5C3C10C18)	0.0; 0.0; 0.0; 0.0; (**3.5**)	0.0; 0.0; 0.0; 0.0; (**1.9**)	0.0; 0.0; 0.0; 0.0; (**2.6**)
(H1C17C9C10)	180.0; 180.0; 180.0; 180.0; (**178.9**)	180.0; 180.0; 180.0; 180.0; (–)	180.0; 180.0; 180.0; 180.0; (**165.0**)
(H10C26C18C10)	180.0; 180.0; 180.0; 180.0; (**178.9**)	180.0; 180.0; 180.0; 180.0; (–)	180.0; 180.0; 180.0; 180.0; (**153.4**)
(H2C18C10C9)	180.0; 180.0; 180.0; 180.0; (**179.4**)	180.0; 180.0; 180.0; 180.0; (–)	180.0; 180.0; 180.0; 180.0; (**180.0**)
(C9C4N1M1)	180.0; 180.0; 180.0; 180.0; (**177.6**)	180.0; 180.0; 180.0; 180.0; (**178.1**)	180.0; 180.0; 180.0; 180.0; (**168.1**)
(C10C3N1M1)	180.0; 180.0; 180.0; 180.0; (**177.5**)	180.0; 180.0; 180.0; 180.0; (**177.9**)	180.0; 180.0; 180.0; 180.0; (**159.9**)
(H1C17C9C4)	0.0; 0.0; 0.0; 0.0; (**3.3**)	0.0; 0.0; 0.0; 0.0; (–)	0.0; 0.0; 0.0; 0.0; (**4.6**)
(H2C18C10C3)	0.0; 0.0; 0.0; 0.0; (**2.1**)	0.0; 0.0; 0.0; 0.0; (–)	0.0; 0.0; 0.0; 0.0; (**7.0**)

* The sign (–) means that there is no corresponding experimental data.

Supplementary Materials

Bonds lengths, valence and torsion angles in the Ni(II), Cu(II) and Zn(II) phthalocyanine metal chelates. The **bold font** in brackets specifies experimental values, regular font, calculated by DFT B3LYP 6-31G(d), OPBE/TZVP, B3PW91/TZVP and wB97XD/TZVP (first, second, third and fourth value, respectively) *.

M	Ni	Cu	Zn
M–N bond lengths, *pm*			
(M1N1)	190.4; 190.3; 191.0; 191.1; (**183.0**)	196.0; 196.2; 196.0; 195.7; (**195.3**)	199.1; 199.6; 199.3; 199.1; (**201.4**)
(M1N2)	190.4; 190.3; 191.0; 191.1; (**183.1**)	195.0; 196.2; 196.0; 195.7; (**195.0**)	199.1; 199.6; 199.3; 199.1; (**201.4**)
(M1N3)	190.4; 190.3; 191.0; 191.1; (**183.0**)	196.0; 196.2; 196.0; 195.7; (**195.3**)	199.1; 199.6; 199.3; 199.1; (**202.0**)
(M1N4)	190.4; 190.3; 191.0; 191.1; (**183.1**)	195.0; 196.2; 196.0; 195.7; (**195.0**)	199.1; 199.6; 199.3; 199.1; (**202.0**)
C–N bond lengths, *pm*			
(N1C3)	138.1; 137.7; 137.1; 136.7; (**137.9**)	137.5; 137.0; 136.6; 136.3; (**138.8**)	137.4; 136.9; 136.5; 136.1; (**136.9**)
(N1C4)	138.1; 137.7; 137.1; 136.7; (**139.0**)	137.6; 137.0; 136.6; 136.3; (**138.9**)	137.4; 136.9; 136.5; 136.1; (**136.6**)
(N2C1)	138.1; 137.7; 137.1; 136.7; (**137.1**)	138.3; 137.0; 136.6; 136.3; (**137.9**)	137.4; 136.9; 136.5; 136.1; (**136.4**)

(N2C2)	138.0; 137.7; 137.1; 136.7; (**137.7**)	138.3; 137.0; 136.6; 136.3; (**138.1**)	137.4; 136.9; 136.5; 136.1; (**136.8**)
(N3C7)	138.1; 137.7; 137.1; 136.7; (**137.9**)	137.5; 137.0; 136.6; 136.3; (**138.8**)	137.4; 136.9; 136.5; 136.1; (**136.5**)
(N3C8)	138.1; 137.7; 137.1; 136.7; (**139.0**)	137.6; 137.0; 136.6; 136.3; (**138.9**)	137.4; 136.9; 136.5; 136.1; (**136.3**)
(N4C5)	138.0; 137.7; 137.1; 136.7; (**139.5**)	138.3; 137.0; 136.6; 136.3; (**137.9**)	137.4; 136.9; 136.5; 136.1; (**136.7**)
(N4C6)	138.1; 137.7; 137.1; 136.7; (**137.7**)	138.3; 137.0; 136.6; 136.3; (**138.1**)	137.4; 136.9; 136.5; 136.1; (**136.3**)
(N5C2)	131.8; 131.5; 131.1; 131.0; (**136.8**)	130.9; 132.3; 131.9; 131.6; (**135.4**)	133.0; 132.9; 132.4; 132.1; (**133.5**)
(N5C3)	131.8; 131.5; 131.1; 131.0; (**137.7**)	135.0; 132.3; 131.9; 131.6; (**137.1**)	133.0; 132.9; 132.4; 132.1; (**134.4**)
(N6C6)	131.8; 131.5; 131.1; 131.0; (**136.8**)	130.9; 132.3; 131.9; 131.6; (**135.4**)	133.0; 132.9; 132.4; 132.1; (**132.9**)
(N6C7)	131.8; 131.5; 131.1; 131.0; (**137.7**)	135.0; 132.3; 131.9; 131.6; (**137.1**)	133.0; 132.9; 132.4; 132.1; (**133.7**)
(N7C4)	131.8; 131.5; 131.1; 131.0; (**138.0**)	134.9; 132.3; 131.9; 131.6; (**134.4**)	133.0; 132.9; 132.4; 132.1; (**133.4**)
(N7C5)	131.8; 131.5; 131.1; 131.0; (**137.3**)	130.8; 132.3; 131.9; 131.6; (**134.9**)	133.0; 132.9; 132.4; 132.1; (**132.9**)
(N8C1)	131.8; 131.5; 131.1; 131.0; (**137.3**)	130.8; 132.3; 131.9; 131.6; (**134.9**)	133.0; 132.9; 132.4; 132.1; (**133.6**)
(N8C8)	131.8; 131.5; 131.1; 131.0; (**138.0**)	134.9; 132.3; 131.9; 131.6; (**134.4**)	133.0; 132.9; 132.4; 132.1; (**133.9**)
C–C bond lengths, *pm*			
(C9C10)	140.0; 139.9; 139.5; 138.9; (**138.3**)	141.2; 140.6; 140.1; 139.5; (**140.7**)	141.0; 141.0; 140.5; 139.9; (**140.1**)
(C11C12)	140.0; 139.9; 139.5; 138.9; (**138.9**)	139.9; 140.6; 140.1; 139.5; (**140.7**)	141.0; 141.0; 140.5; 139.9; (**139.2**)
(C13C14)	140.0; 139.9; 139.5; 138.9; (**138.3**)	141.2; 140.6; 140.1; 139.5; (**140.7**)	141.0; 141.0; 140.5; 139.9; (**140.0**)
(C15C16)	140.0; 139.9; 139.5; 138.9; (**138.9**)	139.9; 140.6; 140.1; 139.5; (**140.7**)	141.0; 141.0; 140.5; 139.9; (**138.4**)
(C9C17)	139.6; 139.5; 139.0; 138.9; (**139.4**)	139.7; 139.4; 138.9; 138.8; (**137.9**)	139.6; 139.4; 138.9; 138.8; (**137.8**)
(C17C25)	139.3; 139.2; 138.7; 138.3; (**139.2**)	139.4; 139.3; 138.7; 138.4; (**137.2**)	139.4; 139.3; 138.8; 138.4; (**138.5**)
(C25C26)	140.9; 140.6; 140.2; 140.2; (**140.7**)	141.0; 140.5; 140.1; 140.1; (**141.2**)	140.8; 140.4; 140.1; 140.0; (**139.6**)
(C26C18)	139.3; 139.2; 138.7; 138.3; (**139.5**)	139.4; 139.3; 138.7; 138.4; (**137.9**)	139.4; 139.3; 138.8; 138.4; (**138.1**)
(C18C10)	139.6; 139.5; 139.0; 138.9; (**138.5**)	139.7; 139.4; 138.9; 138.8; (**137.9**)	139.6; 139.4; 138.9; 138.9; (**138.2**)
C–H bond lengths, *pm*			
(C17H1)	108.5; 108.8; 108.3; 108.2; (**–**)	108.6; 108.8; 108.3; 108.2; (**102.8**)	108.5; 108.9; 108.3; 108.2; (**92.9**)
(C25H9)	108.7; 109.0; 108.4; 108.3; (**–**)	108.7; 109.0; 108.4; 108.3; (**102.3**)	108.7; 109.0; 108.4; 108.3; (**93.0**)
(C26H10)	108.7; 109.0; 108.4; 108.3; (**–**)	108.7; 109.0; 108.4; 108.3; (**102.5**)	108.7; 109.0; 108.4; 108.3; (**93.0**)
(C18H2)	108.5; 108.8; 108.3; 108.2; (**–**)	108.6; 108.8; 108.3; 108.2; (**102.8**)	108.5; 108.9; 108.3; 108.2; (**93.0**)

∠NMN bond angles in the MN_4 chelate node, *deg*			
(N1M1N4)	90.0; 90.0; 90.0; 90.0; (**89.3**)	90.0; 90.0; 90.0; 90.0; (**89.0**)	90.0; 90.0; 90.0; 90.0; (**87.9**)
(N4M1N3)	90.0; 90.0; 90.0; 90.0; (**90.7**)	90.0; 90.0; 90.0; 90.0; (**91.0**)	90.0; 90.0; 90.0; 90.0; (**87.6**)
(N3M1N2)	90.0; 90.0; 90.0; 90.0; (**89.3**)	90.0; 90.0; 90.0; 90.0; (**89.0**)	90.0; 90.0; 90.0; 90.0; (**88.0**)
(N2M1N1)	90.0; 90.0; 90.0; 90.0; (**90.7**)	90.0; 90.0; 90.0; 90.0; (**91.0**)	90.0; 90.0; 90.0; 90.0; (**88.2**)
VAS	360.0; 360.0; 360.0; 360.0; (**360.0**)	360.0; 360.0; 360.0; 360.0; (**360.0**)	360.0; 360.0; 360.0; 360.0; (**351.9**)
∠NNN non-bond angles in the MN_4 chelate node, *deg*			
(N1N4N3)	90.0; 90.0; 90.0; 90.0; (**90.2**)	90.2; 90.0; 90.0; 90.0; (**90.1**)	90.0; 90.0; 90.0; 90.0; (**89.8**)
(N4N3N2)	90.0; 90.0; 90.0; 90.0; (**89.8**)	89.8; 90.0; 90.0; 90.0; (**89.9**)	90.0; 90.0; 90.0; 90.0; (**90.2**)
(N3N2N1)	90.0; 90.0; 90.0; 90.0; (**90.2**)	90.2; 90.0; 90.0; 90.0; (**90.1**)	90.0; 90.0; 90.0; 90.0; (**89.8**)
(N2N1N4)	90.0; 90.0; 90.0; 90.0; (**89.8**)	89.8; 90.0; 90.0; 90.0; (**89.9**)	90.0; 90.0; 90.0; 90.0; (**90.2**)
NVAS	360.0; 360.0; 360.0; 360.0; (**360.0**)	360.0; 90.0; 360.0; 360.0; (**360.0**)	360.0; 360.0; 360.0; 360.0; (**360.0**)
Bond angles in the 6-numbered ring (M1N1C4N7C5N4), *deg*			
(N1M1N4)	90.0; 90.0; 90.0; 90.0; (**89.3**)	90.0; 90.0; 90.0; 90.0; (**89.0**)	90.0; 90.0; 90.0; 90.0; (**87.9**)
(M1N4C5)	126.7; 126.6; 126.4; 126.3; (**130.4**)	125.9; 125.6; 125.5; 125.5; (**127.5**)	125.3; 125.1; 125.1; 125.0; (**124.6**)
(N4C5N7)	127.7; 127.9; 127.7; 127.8; (**126.9**)	128.5; 127.9; 127.7; 127.7; (**127.2**)	127.5; 127.6; 127.5; 127.5; (**128.1**)
(C5N7C4)	121.2; 121.0; 121.8; 121.9; (**116.0**)	122.1; 123.0; 123.5; 123.6; (**122.0**)	124.4; 124.6; 124.9; 125.0; (**123.4**)
(N7C4N1)	127.7; 127.9; 127.7; 127.7; (**126.9**)	127.6; 127.9; 127.7; 127.7; (**126.5**)	127.5; 127.6; 127.5; 127.5; (**127.9**)
(C4N1M1)	126.7; 126.6; 126.4; 126.3; (**130.5**)	125.9; 125.6; 125.5; 125.5; (**127.8**)	125.3; 125.1; 125.0; 125.0; (**124.7**)
VAS[1]	720.0; 720.0; 720.0; 720.0; (**720.0**)	720.0; 720.0; 719.9; 720.0; (**720.0**)	720.0; 720.0; 720.0; 720.0; (**716.6**)
Bond angles in the 5-numbered ring (C3N1C4C9C10), *deg*			
(C3N1C4)	106.5; 106.8; 107.2; 107.4; (**99.9**)	108.0; 108.8; 108.9; 108.9; (**106.1**)	109.4; 109.8; 109.9; 110.0; (**108.8**)
(N1C4C9)	110.5; 110.3; 110.1; 110.0; (**115.9**)	109.6; 109.2; 109.1; 109.1; (**111.4**)	108.8; 108.6; 108.5; 108.4; (**109.1**)
(C4C9C10)	106.3; 106.3; 106.3; 106.3; (**102.6**)	106.4; 106.4; 106.4; 106.4; (**106.5**)	106.5; 106.5; 106.6; 106.6; (**106.1**)
(C9C10C3)	106.3; 106.3; 106.3; 106.3; (**106.5**)	106.4; 106.4; 106.4; 106.5; (**105.5**)	106.5; 106.5; 106.5; 106.6; (**106.7**)
(C10C3N1)	110.4; 110.3; 110.1; 110.0; (**115.1**)	109.6; 109.2; 109.1; 109.1; (**110.4**)	108.8; 108.6; 108.5; 108.4; (**109.3**)
VAS[2]	540.0; 540.0; 540.0; 540.0; (**540.0**)	540.0; 540.0; 540.0; 540.0; (**539.9**)	540.0; 540.0; 540.0; 540.0; (**540.0**)

Bond angles in the 6-numbered ring (C9C10C18C26C25C17), *deg*			
(C9C10C18)	121.4; 121.3; 121.3; 121.4; (**119.9**)	121.0; 121.1; 121.2; 121.3; (**120.0**)	121.1; 121.0; 121.1; 121.2; (**120.4**)
(C10C18C26)	117.4; 117.5; 117.4; 117.3; (**120.9**)	117.8; 117.7; 117.6; 117.5; (**118.0**)	117.7; 117.8; 117.7; 117.6; (**118.4**)
(C18C26C25)	121.2; 121.2; 121.2; 121.3; (**119.0**)	121.2; 121.2; 121.2; 121.2; (**120.7**)	121.2; 121.2; 121.2; 121.2; (**120.7**)
(C26C25C17)	121.2; 121.2; 121.2; 121.3; (**119.9**)	121.2; 121.2; 121.2; 121.2; (**119.7**)	121.2; 121.2; 121.2; 121.1; (**121.4**)
(C25C17C9)	117.4; 117.5; 117.4; 117.3; (**119.9**)	117.8; 117.7; 117.6; 117.5; (**118.5**)	117.7; 117.8; 117.7; 117.7; (**117.7**)
(C17C9C10)	121.4; 121.3; 121.3; 121.4; (**120.4**)	121.0; 121.1; 121.2; 121.3; (**120.8**)	121.1; 121.0; 121.1; 121.2; (**121.4**)
VAS[3]	720.0; 720.0; 719.8; 720.0; (**720.0**)	720.0; 720.0; 720.0; 720.0; (**720.0**)	720.0; 720.0; 720.0; 720.0; (**720.0**)
Selected torsion angles, *deg*			
(M1N1C4N7)	0.0; 0.0; 0.0; 0.0; (**0.0**)	0.0; 0.0; 0.0; 0.0; (**2.7**)	0.0; 0.0; 0.0; 0.0; (**13.4**)
(N1C4C9C17)	180.0; 180.0; 180.0; 180.0; (**180.0**)	180.0; 180.0; 180.0; 180.0; (**172.9**)	180.0; 180.0; 180.0; 180.0; (**179.0**)
(N1C3C10C18)	180.0; 180.0; 180.0; 180.0; (**180.0**)	180.0; 180.0; 180.0; 180.0; (**180.0**)	180.0; 180.0; 180.0; 180.0; (**177.4**)
(N7C4C9C17)	0.0; 0.0; 0.0; 0.0; (**0.0**)	0.0; 0.0; 0.0; 0.0; (**0.0**)	0.0; 0.0; 0.0; 0.0; (**0.2**)
(N5C3C10C18)	0.0; 0.0; 0.0; 0.0; (**0.0**)	0.0; 0.0; 0.0; 0.0; (**0.0**)	0.0; 0.0; 0.0; 0.0; (**3.0**)
(H1C17C9C10)	180.0; 180.0; 180.0; 180.0; (–)	180.0; 180.0; 180.0; 180.0; (**178.5**)	180.0; 180.0; 180.0; 180.0; (**178.3**)
(H10C26C18C10)	180.0; 180.0; 180.0; 180.0; (–)	180.0; 180.0; 180.0; 180.0; (**180.0**)	180.0; 180.0; 180.0; 180.0; (**179.7**)
(H2C18C10C9)	180.0; 180.0; 180.0; 180.0; (–)	180.0; 180.0; 180.0; 180.0; (**180.0**)	180.0; 180.0; 180.0; 180.0; (**179.8**)
(C9C4N1M1)	180.0; 180.0; 180.0; 180.0; (**180.0**)	180.0; 180.0; 180.0; 180.0; (**176.5**)	180.0; 180.0; 180.0; 180.0; (**165.3**)
(C10C3N1M1)	180.0; 180.0; 180.0; 180.0; (**180.0**)	180.0; 180.0; 180.0; 180.0; (**176.3**)	180.0; 180.0; 180.0; 180.0; (**165.1**)
(H1C17C9C4)	0.0; 0.0; 0.0; 0.0; (–)	0.0; 0.0; 0.0; 0.0; (**0.0**)	0.0; 0.0; 0.0; 0.0; (**2.5**)
(H2C18C10C3)	0.0; 0.0; 0.0; 0.0; (–)	0.0; 0.0; 0.0; 0.0; (**0.0**)	0.0; 0.0; 0.0; 0.0; (**2.0**)

* The sign (–) means that there is no corresponding experimental data.

REFERENCES:

Figgis BN, Kucharski ES, Williams GA (1980) Manganese 3d and 4s electron-density distribution in phthalocyaninatomanganese(II). *J Chem Soc Dalton Trans* (9):1515-1525.

Kirner JF, Dow W, Scheidt WR (1976) Molecular stereochemistry of two intermediate-spin complexes. Iron(II) phthalocyanine and manganese(II) phthalocyanine. *Inorg Chem* 15(7):1685-1690.

Reynolds PA, Figgis BN, Kucharski ES, Mason SA (1991) Neutron diffraction at 115 K to 1.09 Å-1 from cobalt phthalocyanine. *Acta Cryst B* 47(6):899-904.

Ballirano P, Caminiti R, Ercolani C, Maras A, Orru MA (1998) X-ray Powder Diffraction Structure Reinvestigation of the α and β Forms of Cobalt Phthalocyanine and Kinetics of the α→β Phase Transition. *J Amer Chem Soc* 120(49):12798-12807.

Jentzen W, Turowska-Tyrk I, Scheidt WR, Shelnutt JA (1996) Planar Solid-State and Solution Structures of (Porphinato)nickel(II) As Determined by X-ray Diffraction and Resonance Raman Spectroscopy. *Inorg Chem* 35(12):3559-3567.

Hoshino A, Takenaka Y, Miyaji H (2003) Redetermination of the crystal structure of α-copper phthalocyanine grown on KCl. *Acta Cryst B* 59(3):393-403.

NBO analysis data

Complex [Co**L1**(O)$_2$]

Alpha occupied eigenvalues (highest) = -5.8373613 eV
Alpha virtual eigenvalues (lowest) = -5.6909715 eV
Beta occupied eigenvalues (highest) = -5.8460685 eV
Beta virtual eigenvalues (lowest) = -5.3225481 eV

<S2>= 0.7609**

Summary of Natural Population Analysis:

Atom	No	Natural Charge	Natural Population Core	Valence	Rydberg	Total
Co	1	0.06473	17.99367	8.91985	0.02174	26.93527
N	2	-0.29894	1.99910	5.27449	0.02535	7.29894
N	3	-0.29053	1.99909	5.26516	0.02627	7.29053
N	4	-0.29384	1.99910	5.26706	0.02769	7.29384
N	5	-0.29052	1.99909	5.26516	0.02627	7.29052
C	6	0.33170	1.99926	3.64805	0.02099	5.66830
C	7	0.33009	1.99926	3.64966	0.02100	5.66991
C	8	0.32872	1.99926	3.65118	0.02083	5.67128
C	9	0.32872	1.99926	3.65118	0.02083	5.67128
C	10	0.33009	1.99926	3.64966	0.02100	5.66991
C	11	0.33170	1.99926	3.64805	0.02099	5.66830
C	12	0.32590	1.99924	3.65338	0.02148	5.67410
C	13	0.32590	1.99924	3.65338	0.02148	5.67410
N	14	-0.35770	1.99937	5.34356	0.01478	7.35770
N	15	-0.35708	1.99936	5.34297	0.01475	7.35708
N	16	-0.35770	1.99937	5.34356	0.01478	7.35770
N	17	-0.35708	1.99936	5.34297	0.01475	7.35708
C	18	-0.21780	1.99911	4.20702	0.01167	6.21780

C	19	-0.21780	1.99911	4.20702	0.01167	6.21780
C	20	-0.21680	1.99910	4.20606	0.01164	6.21680
C	21	-0.21878	1.99910	4.20801	0.01166	6.21878
C	22	-0.21122	1.99911	4.20050	0.01161	6.21122
C	23	-0.21122	1.99911	4.20050	0.01161	6.21122
C	24	-0.21878	1.99910	4.20801	0.01166	6.21878
C	25	-0.21680	1.99910	4.20606	0.01164	6.21680
H	26	0.24501	0.00000	0.75344	0.00155	0.75499
H	27	0.24501	0.00000	0.75344	0.00155	0.75499
H	28	0.24460	0.00000	0.75385	0.00156	0.75540
H	29	0.24452	0.00000	0.75392	0.00156	0.75548
H	30	0.24524	0.00000	0.75321	0.00155	0.75476
H	31	0.24524	0.00000	0.75321	0.00155	0.75476
H	32	0.24452	0.00000	0.75392	0.00156	0.75548
H	33	0.24460	0.00000	0.75385	0.00156	0.75540
O	34	-0.16184	1.99994	6.15611	0.00579	8.16184
O	35	-0.16184	1.99994	6.15611	0.00579	8.16184
* Total *		0.00000	69.97427	132.55353	0.47220	203.00000

NATURAL POPULATIONS: Natural atomic orbital occupancies

NAO	Atom	No	lang	Type(AO)	Occupancy
1	Co	1	S	Cor(1S)	2.00000
2	Co	1	S	Cor(2S)	2.00000
3	Co	1	S	Cor(3S)	1.99638
4	Co	1	S	Val(4S)	0.32018
5	Co	1	S	Ryd(5S)	0.00182
6	Co	1	S	Ryd(6S)	0.00048
7	Co	1	px	Cor(2p)	2.00000
8	Co	1	px	Cor(3p)	1.99882
9	Co	1	px	Val(4p)	0.28745
10	Co	1	px	Ryd(5p)	0.00078
11	Co	1	py	Cor(2p)	2.00000
12	Co	1	py	Cor(3p)	1.99880
13	Co	1	py	Val(4p)	0.28544

14	Co	1	py	Ryd(5p)	0.00080
15	Co	1	pz	Cor(2p)	2.00000
16	Co	1	pz	Cor(3p)	1.99968
17	Co	1	pz	Val(4p)	0.28726
18	Co	1	pz	Ryd(5p)	0.00292
19	Co	1	dxy	Val(3d)	1.98796
20	Co	1	dxy	Ryd(4d)	0.00270
21	Co	1	dxy	Ryd(5d)	0.00041
22	Co	1	dxz	Val(3d)	1.71518
23	Co	1	dxz	Ryd(4d)	0.00483
24	Co	1	dxz	Ryd(5d)	0.00015
25	Co	1	dyz	Val(3d)	1.49330
26	Co	1	dyz	Ryd(4d)	0.00077
27	Co	1	dyz	Ryd(5d)	0.00010
28	Co	1	dx2y2	Val(3d)	1.22011
29	Co	1	dx2y2	Ryd(4d)	0.00167
30	Co	1	dx2y2	Ryd(5d)	0.00002
31	Co	1	dz2	Val(3d)	1.32299
32	Co	1	dz2	Ryd(4d)	0.00426
33	Co	1	dz2	Ryd(5d)	0.00002

Complex [Co**L2**(O)$_2$]

Alpha occupied eigenvalues (highest) = -5.4722031 eV
Alpha virtual eigenvalues (lowest) = -5.5913829 eV
Beta occupied eigenvalues (highest) = -5.4757404 eV
Beta virtual eigenvalues (lowest) = -5.2196943 eV

<S2>= 0.7627**

Summary of Natural Population Analysis:

Atom	No	Natural Charge	Natural Population Core	Valence	Rydberg	Total
Co	1	0.08589	17.99368	8.89943	0.02100	26.91411
N	2	-0.26994	1.99913	5.24462	0.02619	7.26994
N	3	-0.29823	1.99910	5.27169	0.02743	7.29823
N	4	-0.26994	1.99913	5.24462	0.02619	7.26994
N	5	-0.30648	1.99911	5.28230	0.02508	7.30648
C	6	0.32405	1.99924	3.65511	0.02160	5.67595
C	7	0.32405	1.99924	3.65511	0.02160	5.67595
C	8	0.36302	1.99919	3.61739	0.02040	5.63698
C	9	0.36159	1.99919	3.61884	0.02039	5.63841
C	10	0.32877	1.99926	3.65107	0.02091	5.67123
C	11	0.32877	1.99926	3.65107	0.02091	5.67123
C	12	0.36158	1.99919	3.61884	0.02039	5.63842
C	13	0.36302	1.99919	3.61739	0.02040	5.63698
N	14	-0.37149	1.99935	5.35750	0.01464	7.37149
N	15	-0.37317	1.99936	5.35914	0.01468	7.37317
N	16	-0.37318	1.99936	5.35914	0.01468	7.37318
N	17	-0.37149	1.99935	5.35750	0.01464	7.37149
C	18	-0.07684	1.99904	4.06216	0.01563	6.07684
C	19	-0.07582	1.99904	4.06112	0.01565	6.07582
C	20	-0.21542	1.99911	4.20463	0.01168	6.21542
C	21	-0.21542	1.99911	4.20462	0.01168	6.21542

```
      C   22   -0.07581      1.99904     4.06112    0.01565     6.07581
      C   23   -0.07684      1.99904     4.06216    0.01563     6.07684
      C   24   -0.22234      1.99911     4.21150    0.01174     6.22234
      C   25   -0.22234      1.99911     4.21150    0.01174     6.22234
      C   26   -0.17334      1.99913     4.16238    0.01183     6.17334
      C   27   -0.17350      1.99913     4.16254    0.01183     6.17350
      C   28   -0.17350      1.99913     4.16254    0.01183     6.17350
      C   29   -0.17334      1.99913     4.16239    0.01183     6.17334
      C   30   -0.20593      1.99926     4.19488    0.01179     6.20593
      H   31    0.23873      0.00000     0.75944    0.00183     0.76127
      C   32   -0.20552      1.99926     4.19448    0.01178     6.20552
      H   33    0.23881      0.00000     0.75935    0.00184     0.76119
      C   34   -0.20552      1.99926     4.19448    0.01178     6.20552
      H   35    0.23881      0.00000     0.75935    0.00184     0.76119
      C   36   -0.20593      1.99926     4.19488    0.01179     6.20593
      H   37    0.23873      0.00000     0.75944    0.00183     0.76127
      H   38    0.22447      0.00000     0.77430    0.00123     0.77553
      H   39    0.22445      0.00000     0.77431    0.00123     0.77555
      H   40    0.22445      0.00000     0.77431    0.00123     0.77555
      H   41    0.22447      0.00000     0.77430    0.00123     0.77553
      H   42    0.24319      0.00000     0.75521    0.00159     0.75681
      H   43    0.24319      0.00000     0.75521    0.00159     0.75681
      H   44    0.24296      0.00000     0.75545    0.00159     0.75704
      H   45    0.24296      0.00000     0.75545    0.00159     0.75704
      O   46   -0.16732      1.99994     6.16176    0.00562     8.16732
      O   47   -0.16732      1.99994     6.16176    0.00562     8.16732
 =======================================================================
   * Total *    0.00000     85.96734   168.44781    0.58486   255.00000

NATURAL POPULATIONS:  Natural atomic orbital occupancies

   NAO  Atom  No  lang   Type(AO)    Occupancy
 --------------------------------------------
     1   Co    1  S      Cor( 1S)     2.00000
     2   Co    1  S      Cor( 2S)     2.00000
     3   Co    1  S      Cor( 3S)     1.99635
     4   Co    1  S      Val( 4S)     0.31812
```

5	Co	1	S	Ryd(5S)	0.00155
6	Co	1	S	Ryd(6S)	0.00053
7	Co	1	px	Cor(2p)	2.00000
8	Co	1	px	Cor(3p)	1.99885
9	Co	1	px	Val(4p)	0.28233
10	Co	1	px	Ryd(5p)	0.00054
11	Co	1	py	Cor(2p)	2.00000
12	Co	1	py	Cor(3p)	1.99882
13	Co	1	py	Val(4p)	0.28367
14	Co	1	py	Ryd(5p)	0.00079
15	Co	1	pz	Cor(2p)	2.00000
16	Co	1	pz	Cor(3p)	1.99968
17	Co	1	pz	Val(4p)	0.28444
18	Co	1	pz	Ryd(5p)	0.00294
19	Co	1	dxy	Val(3d)	1.98829
20	Co	1	dxy	Ryd(4d)	0.00262
21	Co	1	dxy	Ryd(5d)	0.00043
22	Co	1	dxz	Val(3d)	1.70858
23	Co	1	dxz	Ryd(4d)	0.00478
24	Co	1	dxz	Ryd(5d)	0.00014
25	Co	1	dyz	Val(3d)	1.49798
26	Co	1	dyz	Ryd(4d)	0.00078
27	Co	1	dyz	Ryd(5d)	0.00010
28	Co	1	dx2y2	Val(3d)	1.21593
29	Co	1	dx2y2	Ryd(4d)	0.00154
30	Co	1	dx2y2	Ryd(5d)	0.00003
31	Co	1	dz2	Val(3d)	1.32008
32	Co	1	dz2	Ryd(4d)	0.00420
33	Co	1	dz2	Ryd(5d)	0.00002

Complex [Co**L3**(O)2]

Alpha occupied eigenvalues (highest) = -4.2050334 eV
Alpha virtual eigenvalues (lowest) = -3.6110391 eV
Beta occupied eigenvalues (highest) = -3.8194677 eV
Beta virtual eigenvalues (lowest) = -4.9935792 eV

<S2>= 3.7715**

Summary of Natural Population Analysis:

Atom	No	Natural Charge	Natural Population Core	Valence	Rydberg	Total
Co	1	0.08807	17.99308	8.89610	0.02274	26.91193
N	2	-0.26688	1.99913	5.24069	0.02706	7.26688
N	3	-0.26688	1.99913	5.24069	0.02706	7.26688
N	4	-0.26689	1.99913	5.24069	0.02706	7.26689
N	5	-0.26689	1.99913	5.24069	0.02706	7.26689
C	6	0.40967	1.99920	3.57027	0.02086	5.59033
C	7	0.40966	1.99920	3.57027	0.02086	5.59034
C	8	0.40967	1.99920	3.57027	0.02086	5.59033
C	9	0.40966	1.99920	3.57027	0.02086	5.59034
C	10	0.40967	1.99920	3.57027	0.02086	5.59033
C	11	0.40966	1.99920	3.57027	0.02086	5.59034
C	12	0.40967	1.99920	3.57027	0.02086	5.59033
C	13	0.40966	1.99920	3.57027	0.02086	5.59034
N	14	-0.40137	1.99935	5.38684	0.01518	7.40137
N	15	-0.40137	1.99935	5.38685	0.01518	7.40137
N	16	-0.40137	1.99935	5.38684	0.01518	7.40137
N	17	-0.40137	1.99935	5.38684	0.01518	7.40137
C	18	-0.08239	1.99903	4.06761	0.01574	6.08239
C	19	-0.08239	1.99903	4.06761	0.01574	6.08239
C	20	-0.08239	1.99903	4.06761	0.01574	6.08239
C	21	-0.08239	1.99903	4.06761	0.01574	6.08239
C	22	-0.08239	1.99903	4.06761	0.01574	6.08239
C	23	-0.08239	1.99903	4.06761	0.01574	6.08239
C	24	-0.08239	1.99903	4.06761	0.01574	6.08239

C	25	-0.08239	1.99903	4.06761	0.01574	6.08239
C	26	-0.16186	1.99913	4.15089	0.01184	6.16186
C	27	-0.16186	1.99913	4.15089	0.01184	6.16186
C	28	-0.16186	1.99913	4.15089	0.01184	6.16186
C	29	-0.16186	1.99913	4.15089	0.01184	6.16186
C	30	-0.16186	1.99913	4.15089	0.01184	6.16186
C	31	-0.16186	1.99913	4.15089	0.01184	6.16186
C	32	-0.16186	1.99913	4.15089	0.01184	6.16186
C	33	-0.16186	1.99913	4.15089	0.01184	6.16186
C	34	-0.20363	1.99926	4.19265	0.01172	6.20363
H	35	0.23807	0.00000	0.76007	0.00186	0.76193
C	36	-0.20363	1.99926	4.19265	0.01172	6.20363
H	37	0.23807	0.00000	0.76007	0.00186	0.76193
C	38	-0.20363	1.99926	4.19265	0.01172	6.20363
H	39	0.23807	0.00000	0.76007	0.00186	0.76193
C	40	-0.20363	1.99926	4.19265	0.01172	6.20363
H	41	0.23807	0.00000	0.76007	0.00186	0.76193
C	42	-0.20363	1.99926	4.19265	0.01172	6.20363
H	43	0.23807	0.00000	0.76007	0.00186	0.76193
C	44	-0.20363	1.99926	4.19265	0.01172	6.20363
H	45	0.23807	0.00000	0.76007	0.00186	0.76193
C	46	-0.20363	1.99926	4.19265	0.01172	6.20363
H	47	0.23807	0.00000	0.76007	0.00186	0.76193
C	48	-0.20363	1.99926	4.19265	0.01172	6.20363
H	49	0.23807	0.00000	0.76007	0.00186	0.76193
H	50	0.22503	0.00000	0.77373	0.00124	0.77497
H	51	0.22503	0.00000	0.77373	0.00124	0.77497
H	52	0.22503	0.00000	0.77373	0.00124	0.77497
H	53	0.22503	0.00000	0.77373	0.00124	0.77497
H	54	0.22503	0.00000	0.77373	0.00124	0.77497
H	55	0.22503	0.00000	0.77373	0.00124	0.77497
H	56	0.22503	0.00000	0.77373	0.00124	0.77497
H	57	0.22503	0.00000	0.77373	0.00124	0.77497
O	58	-0.40709	1.99995	6.40267	0.00447	8.40709
O	59	-0.40713	1.99995	6.40271	0.00447	8.40713
* Total *		0.00000	101.95990	204.33340	0.70671	307.00000

NATURAL POPULATIONS: Natural atomic orbital occupancies

NAO	Atom	No	lang	Type(AO)	Occupancy
1	Co	1	S	Cor(1S)	2.00000
2	Co	1	S	Cor(2S)	2.00000
3	Co	1	S	Cor(3S)	1.99573
4	Co	1	S	Val(4S)	0.29892
5	Co	1	S	Ryd(5S)	0.00127
6	Co	1	S	Ryd(6S)	0.00056
7	Co	1	px	Cor(2p)	2.00000
8	Co	1	px	Cor(3p)	1.99873
9	Co	1	px	Val(4p)	0.27569
10	Co	1	px	Ryd(5p)	0.00047
11	Co	1	py	Cor(2p)	2.00000
12	Co	1	py	Cor(3p)	1.99873
13	Co	1	py	Val(4p)	0.27569
14	Co	1	py	Ryd(5p)	0.00047
15	Co	1	pz	Cor(2p)	2.00000
16	Co	1	pz	Cor(3p)	1.99990
17	Co	1	pz	Val(4p)	0.28672
18	Co	1	pz	Ryd(5p)	0.00287
19	Co	1	dxy	Val(3d)	1.98502
20	Co	1	dxy	Ryd(4d)	0.00265
21	Co	1	dxy	Ryd(5d)	0.00046
22	Co	1	dxz	Val(3d)	1.71847
23	Co	1	dxz	Ryd(4d)	0.00409
24	Co	1	dxz	Ryd(5d)	0.00013
25	Co	1	dyz	Val(3d)	1.71847
26	Co	1	dyz	Ryd(4d)	0.00409
27	Co	1	dyz	Ryd(5d)	0.00013
28	Co	1	dx2y2	Val(3d)	1.04555
29	Co	1	dx2y2	Ryd(4d)	0.00125
30	Co	1	dx2y2	Ryd(5d)	0.00003
31	Co	1	dz2	Val(3d)	1.29156
32	Co	1	dz2	Ryd(4d)	0.00424
33	Co	1	dz2	Ryd(5d)	0.00002
